# Differential associations of arousals and respiratory events on oxygen desaturation and resaturation parameters in sleep

**DOI:** 10.1007/s11325-026-03797-7

**Published:** 2026-09-11

**Authors:** Timothy P. Howarth, Tuomas Karhu, Helmi Ben Saad, Ahmed Bahammam, Mieli Luukinen, Purbanka Pahari, Serajeddin Ebrahimian

**Affiliations:** 1 Faculty of Health, Charles Darwin University, Darwin, Australia; 2 Darwin Respiratory and Sleep Health, Darwin Private Hospital, Darwin, Australia; 3 Department of Technical Physics, University of Eastern Finland, Kuopio, Finland; 4 Diagnostic Imaging Center, Kuopio University Hospital, Kuopio, Finland; 5 Faculty of Medicine of Sousse, Farhat HACHED Hospital, Heart Failure (LR12SP09) Research Laboratory, University of Sousse, Sousse, Tunisia; 6 Department of Medicine, University Sleep Disorders Center and Pulmonary Service, King Saud University, Riyadh, Saudi Arabia; 7 The Strategic Technologies Program of the National Plan for Sciences and Technology and Innovation in the Kingdom of Saudi Arabia, Riyadh, Saudi Arabia; 8 Department of Psychiatry, Population Health and Neurology, NYU Grossman School of Medicine, New York, NY USA; 9 Institute of Excellence in Health Equity, Department of Population Health, NYU Grossman School of Medicine, New York, NY USA

**Keywords:** Arousal, Cardiopulmonary physiology, Cardiorespiratory comorbidities, Polysomnography, Sleep apnoea syndromes, Time factors

## Abstract

**Background:**

The severity of nocturnal blood oxygen saturation (SpO_2_) is associated with age, sex, and cardiorespiratory comorbidities. Frequency and timing of nocturnal arousals and respiratory events also display age, sex, and comorbidity-based differences. Hence, it is plausible that resaturation parameters may present an alternative avenue for the assessment of nocturnal arousals.

**Methods:**

First visit data from the Sleep Heart Health Study was utilised to derive SpO_2_ parameters from 5804 participants. Participants missing SpO_2_ parameters, or with no desaturation events were excluded from final analysis. Manually scored arousals were linked to automatically scored SpO_2_ events and defined temporally as pre-event (arousal terminated ≤ 10 s prior to desaturation onset) or within-event (arousal began within the desaturation event or ≤ 5 s after desaturation offset).

**Results:**

5,484 participants (49% female, median age 63 years) were included with 235,996 saturation events. 1.2% of events had pre-event arousals (*n* = 2,850), while 47.6% had within-event arousals (*n* = 112,271). Pre-event arousals were associated with faster desaturation rates (standardized effect: 0.54–0.58) and shorter desaturation durations (by 25–36%). Within-event arousals were associated with lower nadirs, longer desaturation durations, and larger desaturation areas (standardized effects: 0.24–0.51). Both arousal types increased resaturation rates, but through different mechanisms: pre-event arousals shortened resaturation durations while within-event arousals increased resaturation height. These effects were amplified with respiratory events, with the strongest associations observed during apnoeas.

**Conclusions:**

Arousals significantly influence SpO_2_ event architecture, with differential changes seen between desaturation and resaturation parameters.

**Supplementary Information:**

The online version contains supplementary material available at https://doi.org/10.1007/s11325-026-03797-7.

## Introduction

Nocturnal blood oxygen desaturation metrics have shown significant associations with adverse outcomes stemming from obstructive sleep apnoea (OSA) [1–8]. These metrics range from simple counts of occurrence of desaturation events to more complex temporal models, which are assumed to better display the downstream hypoxemic effects associated with apnoeas and hypopnoeas [9]. Desaturation metrics have typically focussed upon the desaturation event as a whole, or only the desaturated ‘half’ of a desaturation event [9]. However, the recovery ‘half’ of a desaturation event (i.e. the “Resaturation”) also displays significant associations with acute outcomes [10] (such as daytime sleepiness assessed via multiple sleep latency test), presence of comorbidities [11] (such as arterial hypertension, heart disease, and stroke) and sleep stages [12], independent of the desaturation side’s parameters. Furthermore, there are significant differences in resaturation parameters and how they change over time based on sex, age, and body mass index (BMI) [10, 13]. Taken together, this suggests that resaturation parameters should be considered as vital metrics for the personalisation of sleep medicine [14].

Although desaturation and resaturation parameters show significant associations with OSA sequelae and comorbidities, they are by no means the only sleep-based parameters associated with sleep disorders [15]. Sleep arousals – defined as abrupt shifts in electroencephalogram (EEG) frequency – are also significantly associated with both short and long-term OSA-related outcomes [16]. Among patients with OSA and particularly severe OSA, the arousal burden is higher compared to non-OSA patients, resulting in significantly more fragmented, discontinuous sleep [17]. Arousals may be preceded by a variety of stimuli, including disruptive respiratory events such as apnoeas or hypopnoeas, abrupt limb movements, external and/or environmental stimuli, or particular chemical thresholds being exceeded [16, 18, 19]. There is, thus, a significant interplay between arousals and desaturation parameters [20]. In addition, airflow increases resulting from ventilatory responses to arousal contribute to restoring blood oxygen levels and reducing excess carbon dioxide (CO_2_) in the blood [16]. On the other hand, chemoreceptor activations due to hypoxia and hypercapnia can contribute to elicit arousal [19], and the association of lower blood oxygen saturation (SpO_2_) nadirs with arousal occurrence has been demonstrated previously [21]. However, no studies have examined the effect of arousals on resaturation parameters or vice versa. Given that both resaturation parameters and the threshold for arousal differ between sexes, and are modulated by age and BMI, it is plausible that resaturation parameters represent another avenue to evaluate the presence of arousals [22].

The use of limited channel sleep studies and home oximeter studies for the assessment of OSA is increasing, and fills a vital healthcare gap for populations with poor access to healthcare [23]. Research has demonstrated that autonomic arousal detection and cardiorespiratory sleep staging can significantly improve the accuracy of home sleep apnoea tests [24]. Furthermore, automated approaches for detecting subtle non-apnoeic/non-hypopnoeic arousal events from polysomnography recordings have shown promising results [25]. These advancements highlight the potential clinical value of identifying novel biomarkers of arousal from limited channel parameters. Understanding the relationship between arousals and oxygen resaturation patterns could therefore provide an additional avenue for improving diagnostic accuracy in simplified sleep testing environments where traditional EEG-based arousal detection is unavailable.

We hypothesised that the presence and timing of arousals significantly influences oxygen resaturation parameters, with differential effects depending on whether arousals occur before or during desaturation events. Furthermore, we anticipated that these effects would be modulated by the presence and type of respiratory events (apnoeas vs. hypopnoeas). Therefore, in this study, we aimed to characterise the effect of nocturnal arousal presence and timing stratified by respiratory event type on oxygen resaturation parameters.

## Methods

### Dataset

For this retrospective analysis of oxygen saturation event-wise data, first-visit data from the Sleep Heart Health Study (SHHS) was utilised. Further details regarding the setting, implementation, and outcomes of the SHHS are available elsewhere [26, 27]. In brief, 5,804 participants from existing prospective cohort studies aged > 40 years completed a baseline examination between 1995 and 1998. Participants provided information on demographic and clinical factors, including medications and comorbidities. For the purposes of this study, we included age, sex, smoking history, height, weight, BMI, comorbidities, and medications as detailed in our previous report [11]. SpO_2_ was captured via an oximeter (Nonin, Minneapolis, MN) recorded at a sampling rate of 1 Hz.

### Data extraction and derivation

**Fig. 1:**
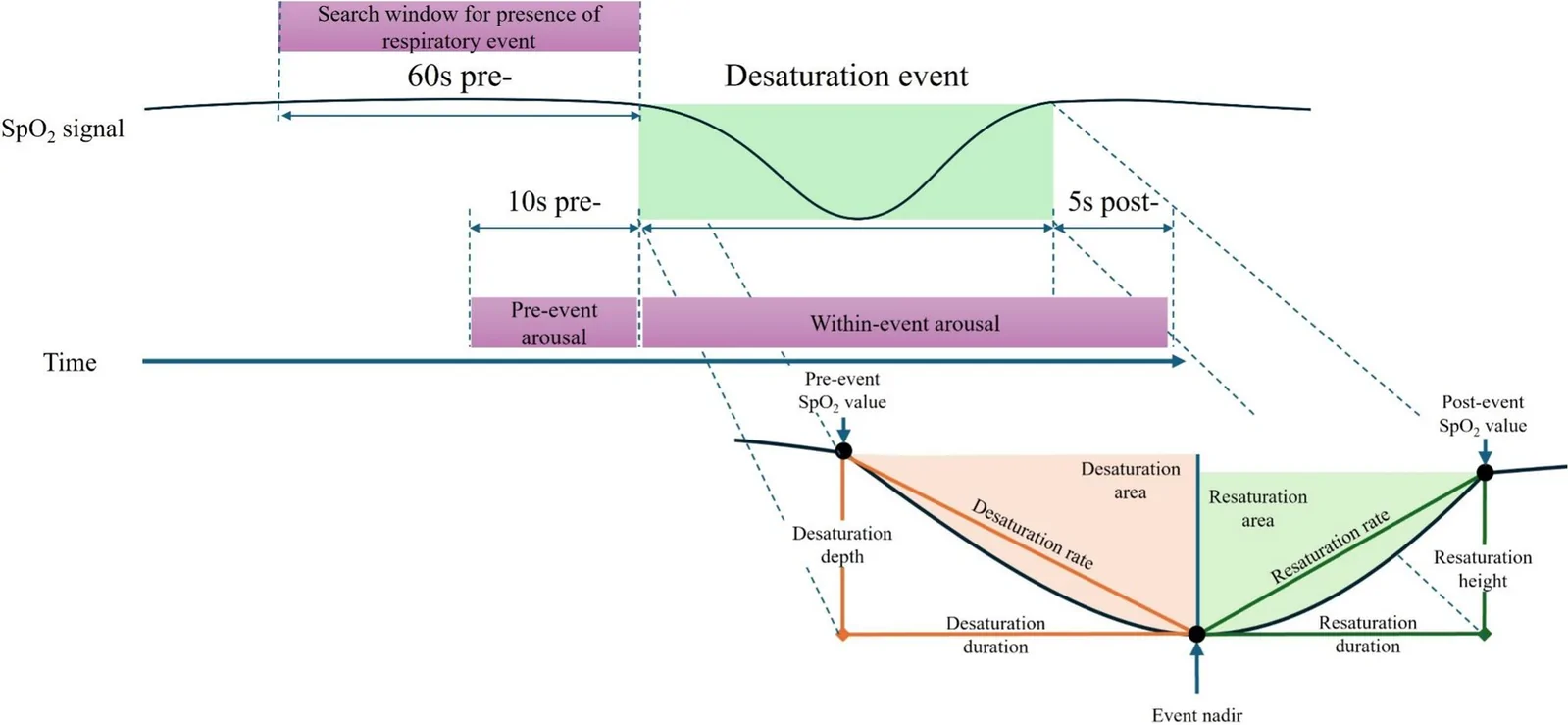
Depiction of the classification method for association with respiratory event, and association with pre-event or within-event arousal. The inset depicts the parameters extracted from ABOSA. **SpO**_**2**_: Oxygen saturation

Due to the known data corruption issues in SHHS data [28] and scoring rules, existing manual labels for desaturation events within polysomnography’s were not used. Instead, European data format files for patients were downloaded and SpO_2_ data analysed utilising the ABOSA (i.e. Automated blood oxygen saturation analysis) software (version 1.1), a freely available automated blood SpO_2_ analysis tool with validated accuracy for detecting and characterizing desaturation events [29, 30]. However, it must be noted that there are currently no formal standards for the assessment of oxygen desaturation events [31]. Therefore, for the ABOSA analyses, the following settings were used: minimum desaturation event duration of five seconds, minimum transient drop 4%, automated artefact removal, and total analysed time defined from sleep onset to offset. From the ABOSA outputs, the following parameters were extracted for analysis: SpO_2_ value at start of desaturation, SpO_2_ value at end of resaturation, duration of desaturations and resaturations (seconds), desaturation depth (percentile point change from event baseline to nadir), and resaturation height (percentile point change from nadir to new baseline), desaturation and resaturation rate (depth (height)/duration) and the desaturation and resaturation area (Fig. 1). Hypnograms were downloaded from the SHHS Profusion files and sleep stages were defined as Wake, N1, N2, N3, and rapid eye movement (REM). The sleep stage during which the event occurred was defined to be the sleep stage in which a desaturation event started, regardless of whether the sleep stage changed during the event. Participants were excluded if they had corrupted or missing nocturnal oximetry data (*n* = 22, 0.4%), or if they recorded no desaturation or resaturation events (*n* = 61, 1.1%). Events that occurred during epochs scored as ‘wake’ were excluded from the analysis (*n* = 58,405, 19.6%), which also resulted in 230 more participants being excluded from the analysis.

Manually annotated arousals did not specify the type of arousal (e.g. respiratory, limb-related, or spontaneous); therefore, no breakdown of arousal type was attempted. To study the association between presence and timing of arousals upon saturation event parameters, in addition to “no arousals”, arousals were divided into two temporal categories: *(i)* Pre-event Arousal – arousals which ended within 10 s prior to the ABOSA calculated start of desaturation event, and *(ii)* Within-event Arousals – arousals which started between the ABOSA defined start of the desaturation event up until 5 s after the ABOSA defined end of resaturation, or 5 s after the defined end of desaturation in the case of events which did not have a resaturation recorded (Fig. 1). As the Pre- and Within-event arousal scoring criteria were non exclusionary (i.e. an arousal could be scored 2 s before desaturation beginning *and* 2 s before resaturation ending), events for which multiple arousals were scored were excluded from the analysis (*n* = 2,444, 1%), which also resulted in seven participants being excluded from analysis. Both sleep staging and arousal definitions were scored according to SHHS rules (found via references [32, 33]).

**Fig. 2:**
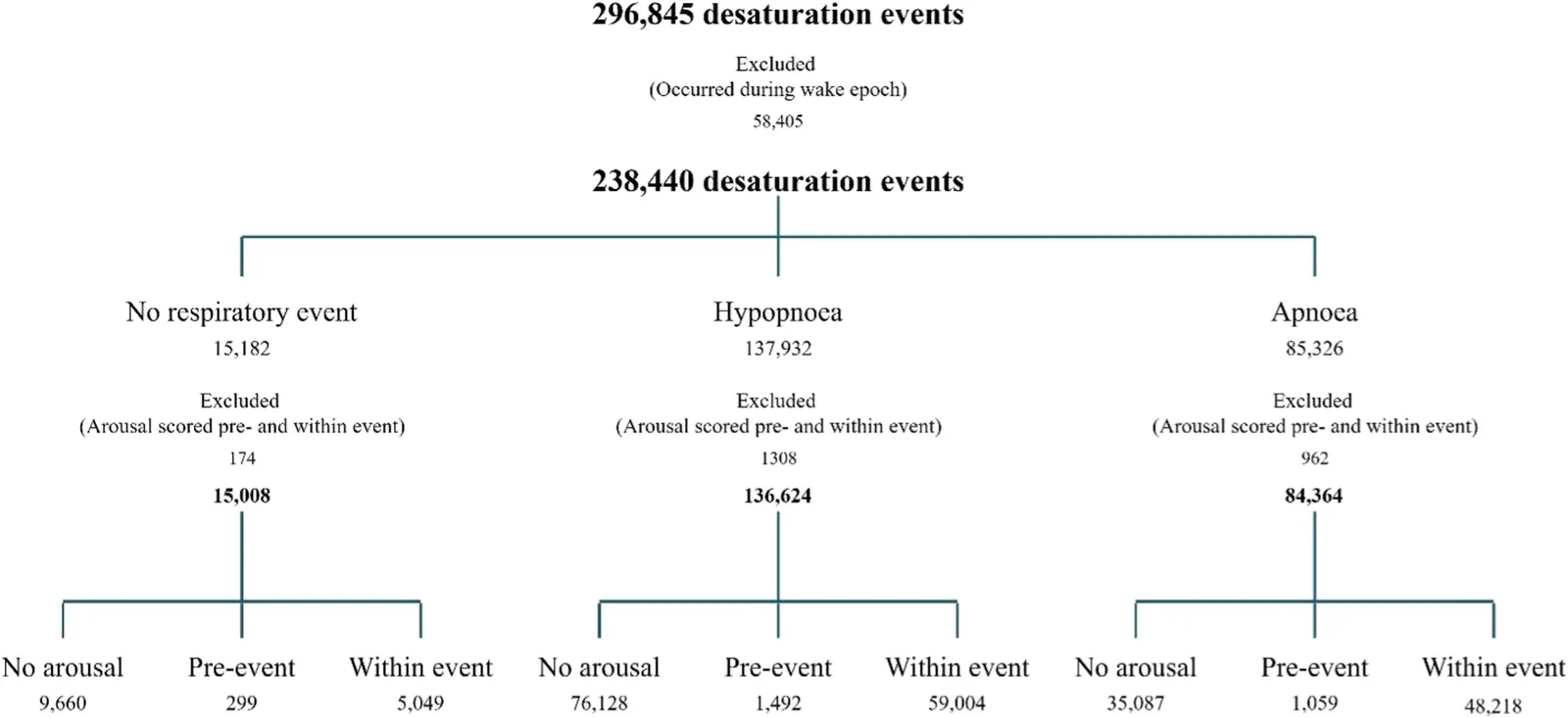
Breakdown of saturation event categories of interaction between respiratory event and arousal timing

Saturation events were additionally categorised by their connection to respiratory events as *(i)* related to hypopnoea - if the ABOSA defined start of the desaturation fell between the start of a hypopnoea until 60 s after the start of a hypopnoea, *(ii)* related to apnoea - if the ABOSA defined start of the desaturation fell between the start of an apnoea until 60 s after the start of an apnoea, or *(iii)* having no related respiratory event (Fig. 2). The 10-second search window prior to desaturation onset and 5-second post-event window for linking arousals align with recent polysomnographic studies of arousal-respiratory event temporal relationships. Leppänen et al. [34] demonstrated that 95% of respiratory event-associated arousals occur within 10 s of event termination. Furthermore, Zitting et al. [35] found that 90% of apnoea-associated cortical arousals initiate within four seconds pre- to nine seconds post-event termination, while hypopnoea-associated arousals occur within six seconds pre- to 14 s post-event. Our selected windows capture this critical period while avoiding overlap with subsequent events in clustered respiratory sequences. Rules for the scoring of respiratory events within the SHHS were: Apnoeas – amplitude of airflow signal decreased by ≥ 75% of baseline (defined as period of stable breathing with stable SpO_2_) for ≥ 10 s; Hypopnoeas - amplitude of airflow signal decreased by ≥ 30% of baseline for ≥ 10 s and for ≥ 2 breaths [36].

### Ethics approval

Initial permissions for the data collection are presented elsewhere [26, 27]. For the present study, National Sleep Research Resource granted the permission to use the data.

### Statistical analysis

Significant differences in event-wise SpO_2_ parameters between males and females were tested via univariate quantile regression for participant-level summary statistics which exhibited skewed distributions. In contrast, mixed effects regression was employed for event-wise analyses to appropriately account for repeated measures within individuals and variability across events. Univariate logistic regression models were utilised to assess differences between males and females in the likelihood of having an SpO_2_ event associated with no respiratory event, hypopnoea or apnoea and no arousal, pre-event arousal or within-event arousal. Associations between presence and timing of arousal (pre-event and within-event against reference of no-arousal, and comparison of pre-event against within-event arousal) were initially assessed via univariate mixed effects regression stratified by no associated respiratory event, hypopnoea and apnoea. Following this, retaining the respiratory event stratification, the association between presence and timing of arousal upon SpO_2_ parameters was assessed in multivariate mixed effects models adjusted for age, sex, BMI, forced expiratory volume in one second (percentage of predicted value utilising ethnic neutral norms via the updated global lung initiative 2023 equations [37]), sleep stage of event, smoking history and presence of arterial hypertension, lung disease, myocardial infarction, diabetes mellitus, stroke, heart failure, use of hormone therapy or mental health medications (covariates chosen on the basis of our previous research [11, 12]). Event nadir and desaturation duration, depth, rate and area were additionally adjusted for pre-event SpO_2_ value, while post-event SpO_2_ value and resaturation duration, height, rate and area were additionally adjusted for pre-event SpO_2_ value and event nadir. All statistical tests were adjusted for via Bonferroni correction, and all analyses conducted in STATA 18 (College station, Texas).

## Results

### Participant characteristics

A total of 5,484 participants (49% female) were included for analysis. Participants were a median 63 years of age (interquartile range (IQR) 55, 72), with the majority being overweight (42.1%) or obese (31.3%) (Supplement 1). Around half (53.5%) of the participants had a history of smoking and the majority of participants had at least one comorbidity (85.7%), of which arterial hypertension was the most common (49.5%). One-third of female participants recorded use of hormone therapy (30%).

Overall, 235,996 desaturation events were recorded (participant median 19 (IQR 6, 50)), though 16,881 (7.1%) desaturation events did not have a corresponding resaturation recorded. Males recorded a significantly higher number of desaturations and resaturations than females (median difference 14 & 13 respectively) and numerous significant differences were seen in SpO_2_ architecture parameters (Table 1). Pre- and post-SpO_2_ values were a median 0.2% lower among females, desaturation and resaturation durations 1.8 & 0.3 s shorter, and the desaturation and resaturation areas 13.1 & 3.4%s smaller than males.

**Table 1:** Desaturation and resaturation parameters for males and females

Parameters	Total (*n* = 5,484)	Female (*n* = 2,799)	Male (*n* = 2,685)	Beta (95% CI)
Number of desaturations (participant median (IQR))	235,996 (19 (6, 50))	88,823 (13 (5, 36))	147,173 (27 (10, 66))*	14 (12.09, 15.91)
Number of resaturations (participant median (IQR))	219,115 (17 (5, 46))	82,284 (12 (4, 33))	136,831 (25 (8, 59))*	13 (11.09, 14.91)
*Percentage of desaturation events with corresponding resaturation*	*92.9%*	*92.6%*	*93.0%*	*-*
Pre-event SpO_2_ value	95.60 (95.58, 95.61)	95.58 (95.56, 95.60)	95.60 (95.59, 95.62)*	−0.17 (−0.26, −0.08)
Saturation event nadir	88.23 (88.21, 88.25)	88.61 (88.58, 88.64)	88.00 (87.98, 88.03)*	−0.33 (−0.45, −0.21)
Post-event SpO_2_ value	95.62 (95.60, 95.63)	95.59 (95.57, 95.61)	95.63 (95.62, 95.65)*	−0.18 (−0.28, −0.08)
Desaturation duration (seconds)	29.10 (29.03, 29.16)	28.20 (28.09, 28.30)	29.64 (29.56, 29.71)*	1.76 (1.34, 2.17)
Desaturation depth (% point change)	7.36 (7.35, 7.38)	6.97 (6.95, 7.00)	7.60 (7.58, 7.62)*	0.19 (0.11, 0.28)
Desaturation rate (%/second)	0.30 (0.30, 0.31)	0.31 (0.31, 0.31)	0.30 (0.30, 0.30)*	−0.03 (−0.03, −0.02)
Desaturation area (%s)	125.94 (125.45, 126.43)	113.78 (113.05, 114.51)	133.28 (132.64, 133.93)*	13.1 (10.53, 15.67)
Resaturation duration (seconds)	13.09 (13.05, 13.12)	12.99 (12.93, 13.05)	13.14 (13.10, 13.19)*	0.34 (0.13, 0.55)
Resaturation depth (% point change)	7.47 (7.45, 7.49)	7.06 (7.03, 7.08)	7.72 (7.69, 7.74)*	0.2 (0.1, 0.29)
Resaturation rate (%/second)	0.70 (0.70, 0.70)	0.68 (0.67, 0.68)	0.72 (0.71, 0.72)	−0.01 (−0.02, 0)
Resaturation area (%s)	48.98 (48.78, 49.18)	45.86 (45.54, 46.17)	50.86 (50.60, 51.12)*	3.43 (2.45, 4.4)

Data displayed as mean (95% CI)

* Indicates Bonferroni adjusted *p*-value < 0.05 obtained via univariate quantile regression (summary parameters) or univariate mixed effects regression between males and females

Beta value obtained from univariate quantile regression (summary parameters) or univariate mixed effects regression, showing difference between female and male participants

Abbreviations: *CI* Confidence interval, *IQR* Interquartile range, *SpO*_2_ Oxygen saturation

In fully adjusted models, differences between males and females in pre- and post-event SpO_2_ reversed (Supplement 2), however differences in duration and area remained, alongside significantly slower desaturation and resaturation rates in males.

### Desaturation event characteristics

220,988 saturation events (93.6%) were associated with either an apnoea (*n* = 84,364, 35.7%) or hypopnoea (*n* = 136,624, 57.9%) (Table 2). Females recorded a significantly higher proportion of events associated with hypopnoeas (63.5%) or no respiratory event (8.9%) compared to males (54.5 & 4.8% respectively). 115,121 saturation events (48.8%) were associated with either a pre-event (*n* = 2,850, 2.5%) or a within-event arousal (*n* = 112,271, 97.5%). Females recorded a significantly higher proportion of pre- or within- event arousals for events with no respiratory event, while males recorded a higher proportion of pre- or within-event arousals in events with associated apnoeas.

**Table 2:** The number of saturation events associated with arousals and respiratory events by sex

Parameters	Total (*n* = 235,996)	Female (*n* = 88,823)	Male (*n* = 147,173)	OR (95% CI)
No respiratory related event	15,008 (6.4%)	7,930 (8.9%)	7,078 (4.8%)*	0.52 (0.5, 0.53)
- No Arousal (*n* (%)) (median (IQR))	9,660 (4.1%) (1 (0, 2))	5,167 (5.8%) (1 (0, 2))	4,493 (3.1%)* (1 (0, 2))	0.51 (0.49, 0.53)
- Arousal pre-event (*n* (%)) (median (IQR))	299 (0.1%) (0 (0, 0))	151 (0.2%) (0 (0, 0))	148 (0.1%)* (0 (0, 0))	0.59 (0.47, 0.74)
- Arousal within-event (*n* (%)) (median (IQR))	5,049 (2.1%) (0 (0, 1))	2,612 (2.9%) (0 (0, 1))	2,437 (1.7%)* (0 (0, 1))	0.56 (0.53, 0.59)
Hypopnoea	136,624 (57.9%)	56,374 (63.5%)	80,250 (54.5%)*	0.69 (0.68, 0.7)
- No Arousal (*n* (%)) (median (IQR))	76,128 (32.3%) (6 (1, 17))	33,921 (38.2%) (4 (1, 14))	42,207 (28.7%)* (7 (2, 20))	0.65 (0.64, 0.66)
- Arousal pre-event (*n* (%)) (median (IQR))	1,492 (0.6%) (0 (0, 0))	571 (0.6%) (0 (0, 0))	921 (0.6%) (0 (0, 0))	0.97 (0.88, 1.08)
- Arousal within-event (*n* (%)) (median (IQR))	59,004 (25%) (4 (1, 13))	21,882 (24.6%) (3 (1, 9))	37,122 (25.2%)* (6 (2, 17))	1.03 (1.01, 1.05)
Apnoea	84,364 (35.7%)	24,519 (27.6%)	59,845 (40.7%)*	1.8 (1.77, 1.83)
- No Arousal (*n* (%)) (median (IQR))	35,087 (14.9%) (1 (0, 5))	11,793 (13.3%) (0 (0, 3))	23,294 (15.8%)* (1 (0, 7))	1.23 (1.2, 1.26)
- Arousal pre-event (*n* (%)) (median (IQR))	1,059 (0.4%) (0 (0, 0))	258 (0.3%) (0 (0, 0))	801 (0.5%)* (0 (0, 0))	1.88 (1.63, 2.16)
- Arousal within-event (*n* (%)) (median (IQR))	48,218 (20.4%) (1 (0, 5))	12,468 (14%) (0 (0, 2))	35,750 (24.3%)* (2 (0, 10))	1.96 (1.92, 2.01)

Parameters displayed as number (%) and median (IQR) per participant

* Bonferroni adjusted *p*-value < 0.05 for effect of sex obtained via univariate logistic regression

Odds ratio (obtained via univariate logistic regression) detailing the odds of male participants recording this type of event combination against the odds of female participants recording this type of event combination

Abbreviations: *CI* Confidence interval, *IQR* Interquartile range, *OR* Odds ratio

### Arousal timing and presence of respiratory events

**Fig. 3:**
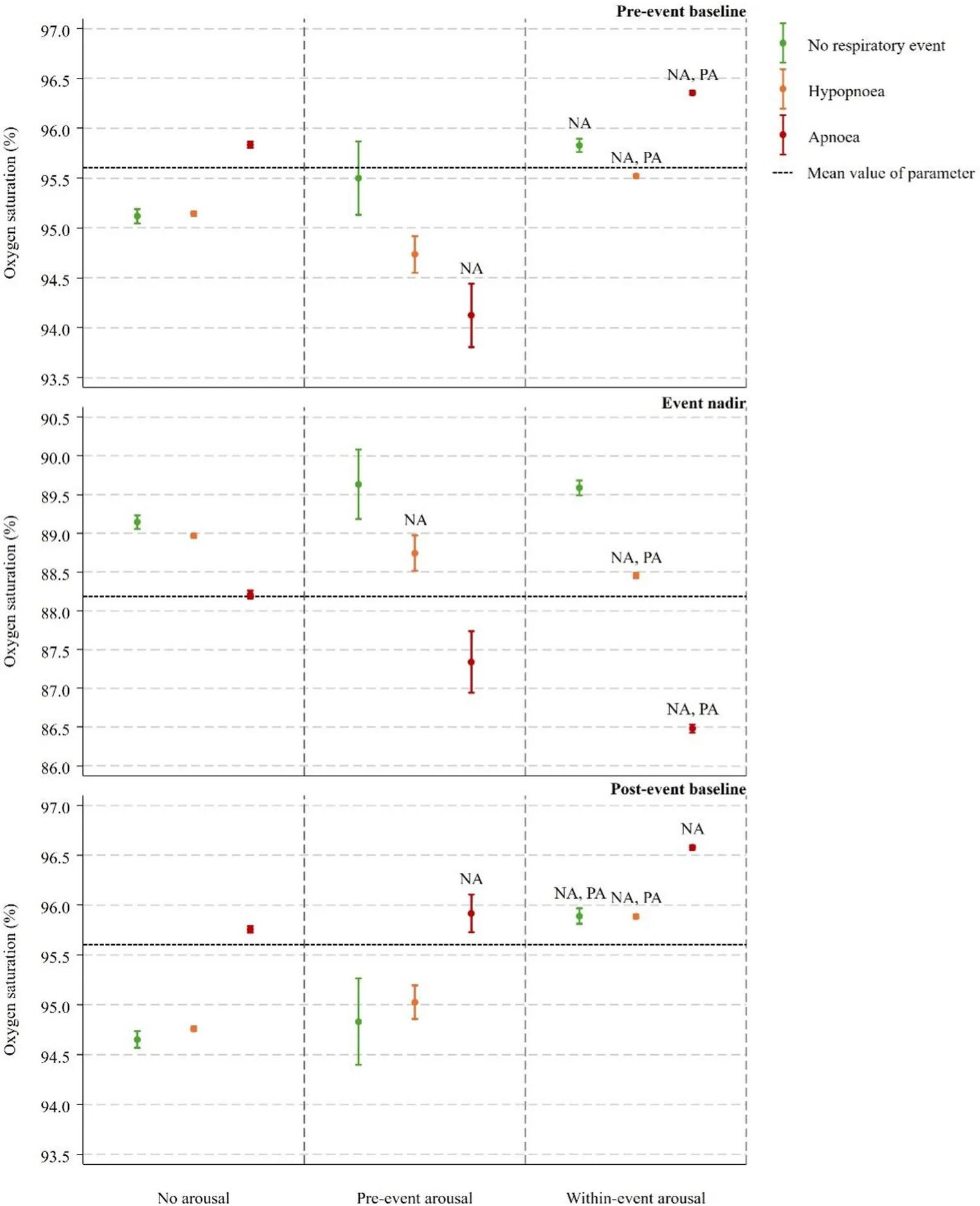
Means (95% confidence intervals) of pre- and post-event baselines and event nadirs by presence and timing of arousal, and presence and type of respiratory event. **NA** indicates Bonferroni corrected p-value < 0.05 compared to No Arousal presence in the same respiratory event category. **PA** indicates Bonferroni corrected p-value < 0.05 compared to Pre-Event Arousal presence in the same respiratory event category

**Fig. 4:**
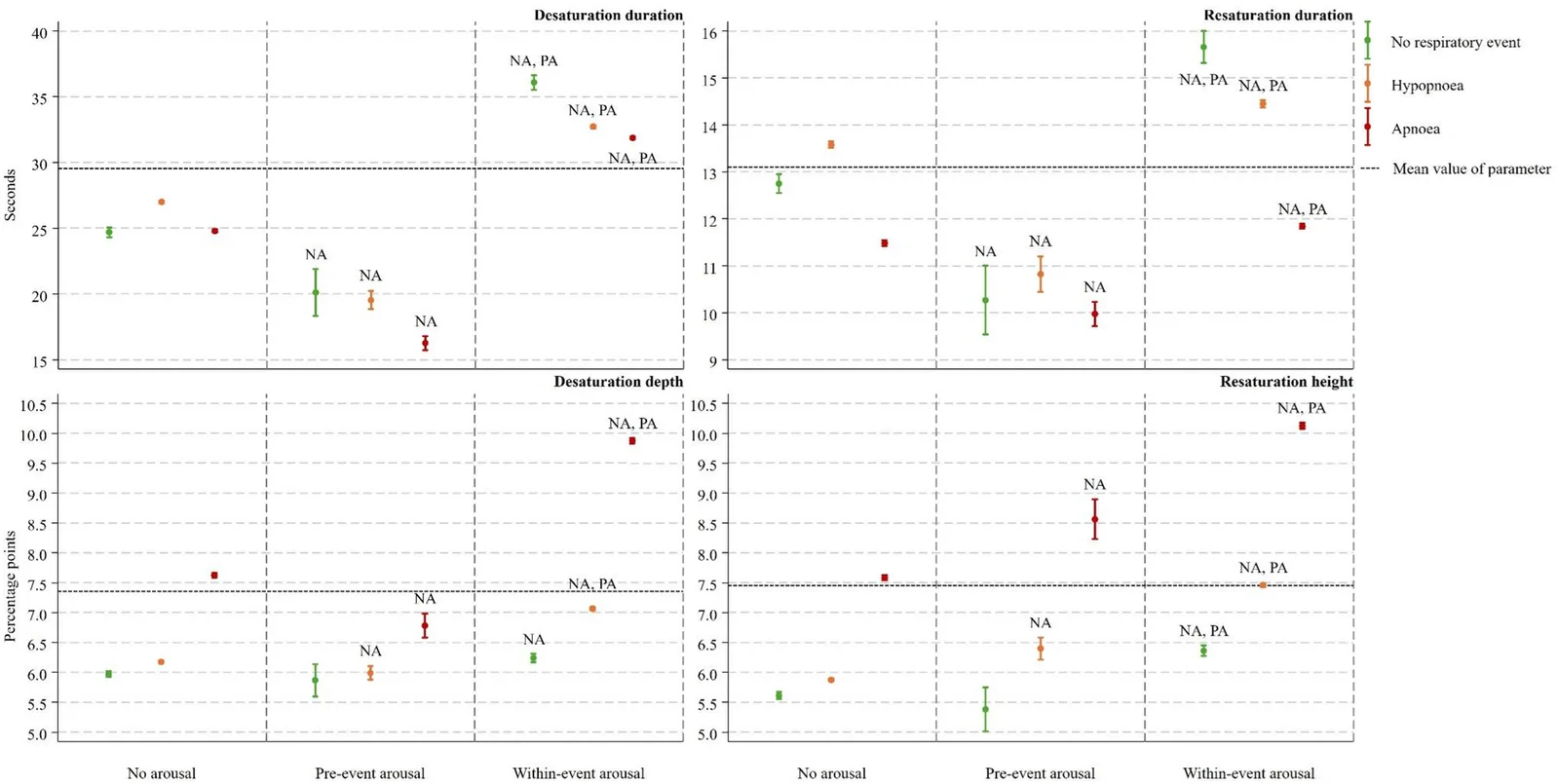
Means (95% confidence intervals) of desaturation and resaturation duration and depth/height by presence and timing of arousal, and presence and type of respiratory event. **NA** indicates Bonferroni corrected p-value < 0.05 compared to No Arousal presence in the same respiratory event category. **PA** indicates Bonferroni corrected p-value < 0.05 compared to Pre-Event Arousal presence in the same respiratory event category

**Fig. 5:**
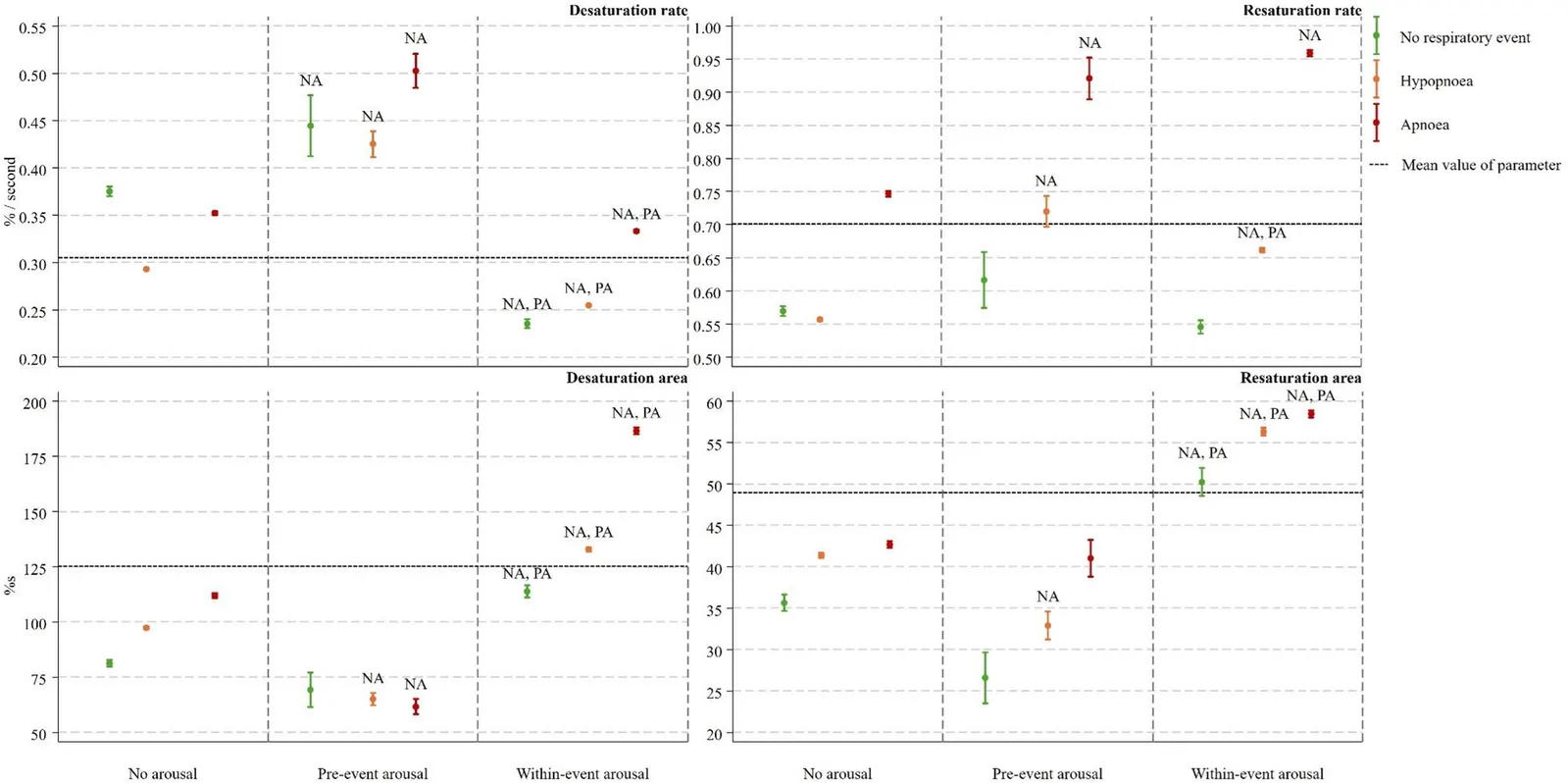
Means (95% confidence intervals) of desaturation and resaturation rate and area by presence and timing of arousal, and presence and type of respiratory event. **NA** indicates Bonferroni corrected p-value < 0.05 compared to No Arousal presence in the same respiratory event category. **PA** indicates Bonferroni corrected p-value < 0.05 compared to Pre-Event Arousal presence in the same respiratory event category

Altogether, regardless of respiratory event presence/type, desaturation events associated with a pre-event arousal were shorter/faster and less severe (judged by area), while those associated with a within-event arousal were longer, slower and more severe (judged by area and nadirs) (Figs. 3, 4 and 5).

### No respiratory event

#### Pre-event arousal vs. no arousal

Pre-event arousals were associated with shorter desaturation and resaturation durations and faster desaturation rates.

#### Within-event arousal vs. no arousal

Within-event arousals were associated with higher pre- and post-event SpO_2_ values, longer desaturation and resaturation durations, greater desaturation depths and resaturation heights, slower desaturation rates and larger desaturation and resaturation areas.

#### Within-event arousal vs. pre-event arousal

Within-event arousals were associated with higher post-event SpO_2_ values, longer desaturation and resaturation durations, larger resaturation heights, slower desaturation rates and larger desaturation and resaturation areas.

### Hypopnoeas

#### Pre-event arousal vs. no arousal

Pre-event arousals were associated with lower event nadirs, shorter desaturation and resaturation durations, smaller desaturation depths yet greater resaturation heights, faster desaturation and resaturation rates and smaller desaturation and resaturation areas.

#### Within-event arousal vs. no arousal

Within-event arousals were associated with higher pre- and post-event SpO_2_ values, lower event nadirs, longer desaturation and resaturation durations, greater desaturation depths and resaturation heights, slower desaturation rates yet faster resaturation rates and larger desaturation and resaturation areas compared.

#### Within-event arousal vs. pre-event arousal

Within-event arousals were associated with higher pre- and post-event SpO_2_ values, lower event nadirs, longer desaturation and resaturation durations, larger desaturation depths and resaturation heights, slower desaturation and resaturation rates and larger desaturation and resaturation areas.

### Apnoeas

#### Pre-event arousal vs. no arousal

Pre-event arousals were associated with lower pre-event SpO_2_ values and higher post-event SpO_2_ values, shorter desaturation and resaturation durations, smaller desaturation depths yet greater resaturation heights, faster desaturation and resaturation rates and smaller desaturation areas.

#### Within-event arousal vs. no arousal

Within-event arousals were associated with higher pre- and post-event SpO_2_ values, lower event nadirs, longer desaturation and resaturation durations, greater desaturation depths and resaturation heights, slower desaturation rates yet faster resaturation rates and larger desaturation and resaturation areas.

#### Within-event arousal vs. pre-event arousal

Within-event arousals were associated with higher pre-event SpO_2_ values, lower event nadirs, longer desaturation and resaturation durations, larger desaturation depths and resaturation heights, slower desaturation rates and larger desaturation and resaturation areas.

### Multivariate regression analysis results

**Fig. 6:**
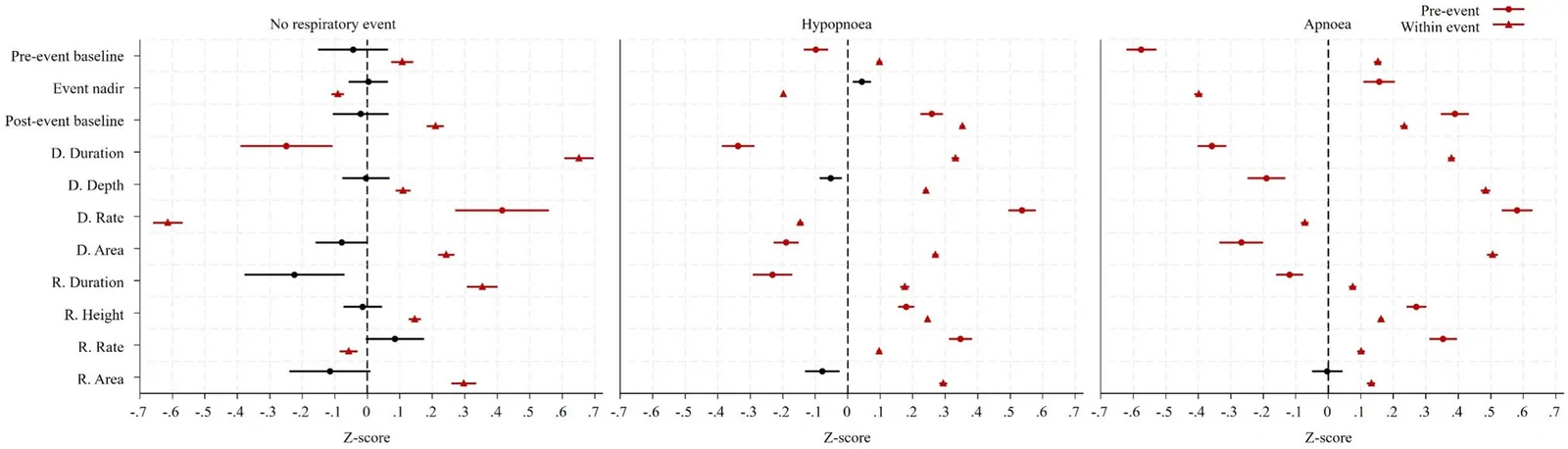
Multivariate mixed effect regression results showing associations between arousal time (reference no arousal) and respiratory event on standardised values of desaturation and resaturation parameters. Models were adjusted for age, sex, body mass index, forced expiratory volume in one second, sleep stage, smoking history, and presence/history of arterial hypertension, lung disease, heart failure, diabetes mellitus, myocardial infarction, stroke, use of hormone therapy and mental health medications. Desaturation duration, depth, rate, and area were additionally adjusted for pre-event baseline oxygen saturation (SpO_2_). Resaturation duration, depth, rate, and area were additionally adjusted for pre-event baseline SpO_2_ and event nadir. **D.**, Desaturation; **R.**, Resaturation

In multivariate mixed effects modelling, the differential associations between timing of arousals and type of respiratory event on desaturation and resaturation parameters remained (Fig. 6).

For saturation events with no associated respiratory event; pre-event arousals in most cases had no significant association with desaturation or resaturation parameters. Yet, within-event arousals were associated with increased pre- and post-event SpO_2_ values, durations, depths (heights), and areas and decreased event nadirs and rates.

For events with an associated respiratory event, the direction of association between saturation parameters and pre-event or within-event arousals was the same for apnoeas and hypopnoeas, however the effect size was greater among those saturation events associated with apnoeas.

In saturation events with an associated respiratory event, contrasting directions of association were noted between pre- and within-event arousals for multiple saturation parameters. Pre-event arousals were associated with lower pre-event SpO_2_ values, shorter desaturation and resaturation durations, higher event nadirs, faster desaturation rates and smaller resaturation areas.

In contrast, within-event arousals were associated with higher pre-event SpO_2_ values, longer desaturation and resaturation durations, lower event nadirs, slower desaturation rates and larger desaturation areas.

Only post-event SpO_2_ values, resaturation height and resaturation rate showed the same direction of association for both pre- and within-event arousals. However the faster resaturation rate associated with a pre-event arousal was significantly faster than that associated with a within-event arousal (hypopnoea: 0.35 (95% confidence interval (CI) 0.31, 0.38) vs. 0.10 (95% CI 0.09, 0.10) and apnoea: 0.35 (95% CI 0.31, 0.40) vs. 0.10 (95% CI 0.09, 0.11)). Additionally, the desaturation rate was reduced significantly more when there was a within-event arousal and no respiratory event (−0.62 (95% CI −0.66, −0.57)), compared to when there was a within-event arousal and either an apnoea (−0.07 (95% CI −0.08, −0.06)) or hypopnoea (−0.15 (95% CI −0.16, −0.14)).

## Discussion

The current study shows significant associations between arousals and respiratory events for both desaturation and resaturation parameters. In line with our previous studies [1, 10, 11, 13], an association with resaturations was identified despite adjustment for desaturation parameters, further solidifying the notion that both halves of a saturation event should be considered separately when estimating the immediate physiological consequences of OSA. The key finding is contrasting associations between arousals and desaturation or resaturation parameters; pre-event arousals were associated with decreased desaturation depth but increased resaturation height, while within-event arousals were associated with a slowed desaturation rate, but an increased resaturation rate. Arousals associated with apnoeas amplified this compared to hypopnoea-associated or non-respiratory arousals and showed a stronger association with desaturation parameters than resaturation parameters (Supplement 3).

A significantly higher number of desaturations was recorded for males than females (median 27 vs. 13) and an overall higher proportion of arousal-associated desaturations, in line with previous literature identifying arousal burden being associated with apnoea-hypopnoea burden. However, in cases where there was no respiratory event recorded, females recorded a higher proportion of arousal-associated desaturations (Table 2). This may reflect a higher burden of limb-related or spontaneous arousals in women, and thus may indicate an alternate pathway resulting in desaturation which is non-respiratory. Additionally, across all conditions of arousal presence and timing, males showed a slower resaturation rate than females (Supplement 2) – contrary to our previous suggestion that the heightened rate of resaturation among females may be due to arousals [11]. Notably, although there was no significant difference in age, BMI or lung function between females and males, a higher proportion of females recorded some form of medication or comorbidity which would be expected to slow the resaturation rate [11]. Prior work has demonstrated sex-specific OSA phenotypes [38], with potential mechanisms underlying this being ventilatory chemosensitivity [39, 40], vascular reactivity [41] or upper-airway structure. Our research indicates there may also be sex specific desaturation architecture with alternate underlying physiological pathways.

The shorter desaturation/resaturation durations and faster rates observed with pre-event arousals in the current study **(**Fig. 6**)** may be explained by arousal-induced upper airway dilator muscle activation persisting across breathing cycles [41, 42]. This sustained activation of upper airway dilator muscles may be protective against subsequent airway collapse [43]. Studies comparing OSA patients and healthy subjects have shown that genioglossus activity during sleep can increase to levels exceeding those during wakefulness, potentially as a compensatory mechanism [44]. While the presence of a respiratory event partially attenuated this association in the current study, it did not do so completely. Thus, non-respiratory arousals may have an influence on desaturation event architecture, with distinct underlying physiological mechanisms.

In contrast, within-event arousals showed almost the opposite profile – being associated with longer desaturation/resaturation durations and a deeper nadir. As our study design was cross-sectional with respect to individual events, and due to the differences in the automated/manual scoring of saturation events/arousals, we cannot establish directionality. However, this pattern is consistent with the arousal threshold endotype where a higher arousal threshold allows a greater hypoxaemic load to accumulate before arousal occurs [45]. Similarly, Huang et al. [46] demonstrated that in patients with very severe OSA, the respiratory arousal index strongly correlates with AHI, mean desaturation, desaturation index, and inversely with mean SpO₂. This supports the interpretation that arousals can be triggered by hypoxemic load accumulating to critical thresholds, accounting for the more severe desaturation profiles seen with within-event arousals. This further supports the utility of implementing fine detail desaturation/resaturation analytics into the clinical workstream when higher level sleep studies are unavailable or unfeasible.

The current AASM definition treats arousals as binary when a multitude of recent research has demonstrated scalable arousal intensity [20, 47–49], and poor interobserver agreement in scoring [50]. Recent work has demonstrated that the magnitude of cardiovascular response to arousal correlates with arousal intensity and the severity of oxygen desaturation events [51, 52] as does the ventilatory response [53]. In the current study, within-event arousals were associated with deeper nadirs and larger desaturation areas compared to desaturation events with no arousal, adding to this body of evidence. Furthermore, the faster resaturation rates we noted following within-event arousals fits with these cardiac and ventilatory responses, on top of a sympathetic surge upon respiratory event termination [54]. Our finding of an increased resaturation duration following within-event arousals may be explained by the biphasic ventilatory response demonstrated by Jordan et al. [55] & Dutta et al. [56], where hypoventilation follows the initial hyperventilation.

The distinct relationships we found between arousal presence and timing and SpO_2_ dynamics could contribute to more sophisticated approaches for detecting sleep disruptions in limited channel settings addressing the growing need in general medicine and critical care for more convenient and accessible sleep apnoea detection, management and follow-up. Pulse oximetry based sleep-wake classification is already feasible [57–59], and could integrate arousal-sensitive saturation event features to extend their capability – though further validation against contemporarily scored arousals is required.

## Limitations

The results of the current study are influenced by the selection of our saturation event thresholds and the temporal thresholds chosen for respiratory and arousal linkage. The 4% desaturation threshold aligns with the 4% ODI used across Europe [60], is associated with cardiovascular disease where lower thresholds are not [61], and is less susceptible to artefacts in automated analyses compared to a 3% threshold. The 5-second minimum desaturation event duration follows the initial ABOSA validation, which recorded a Cohen’s kappa of 0.8–0.9 (dataset dependent) against manual scoring [29]. The 10-second pre-event search window for arousal linkage is short enough to relate to the rapid onset of arousal-associated physiological responses [62], and we extended the within-event window to five seconds beyond event offset in line with prior work [34]. Physiological validation would be required to establish whether they capture the intended temporal relationships. This could involve simultaneous measures of ventilatory or autonomic response, or cohorts with directly measured circulation delay. Yet, the differential time window of the pre- and within- event arousals itself also potentially biases towards a finding of more severe desaturation events. Altogether, due to the delay via lung to finger circulation time plus a potential delay in instrument recording, we cannot be certain if a respiratory event was related to a particular desaturation event, or even if potentially multiple respiratory events were related to a desaturation event. For the same reason, the timing of arousals is defined relative to the oximetric desaturation rather than the underlying physiological event, and with the timeframe and the methods available and used, we cannot be certain if a pre-event arousal (or even a within-event arousal) was directly associated with the desaturation event – e.g. if the arousal stimulated a physiological event which resulted in desaturation, or, if a physiological event caused both the arousal and the desaturation, but with a different time lag until arousal compared to desaturation.

Arousals were manually scored while saturation events were derived from automated analyses raising the possibility of temporal mismatch. Furthermore, we did not assess the exact cause of each arousal, nor the arousal intensity and nor did we adjust for the duration and magnitude of airflow restriction beyond the “apnoea” and “hypopnoea” categorisation which would likely modulate the associations we report. However, distinct arousal types would have different physiological profiles which may result in distinct associations (or lack thereof) upon desaturation event architecture. The stratification by respiratory event type partially mitigates this, as saturation events with no associated respiratory event necessarily can reflect non-respiratory arousals, and the associations observed in this stratum differed markedly from those seen with respiratory events. Furthermore, apnoea/hypopnoea scorings in the SHHS dataset followed the scoring rules of the time which has been updated in the interim years. The pre-event/within-event temporal rules are necessarily arbitrary and do not account for possible chains of desaturation events which may themselves influence saturation event architecture. Events with multiple linked arousals were excluded and although small in number, this may also have biased towards isolated, non-chain events. The 4% threshold will have missed shallower desaturations, and a proportion of events did not have a resaturation side recorded. We excluded events occurring during epochs marked as “Wake”, which accounted for almost 20% of events. This likely reflects both temporal mismatch with manual sleep staging, and the well-known limitations of 30-second epoch scoring. Finer (1–5 s) automated staging may help in future work. Strengths of the study include the SHHS’s comprehensive polysomnographic data with manually scored arousals and respiratory events, the large sample size, community-based sampling, and broad demographic inclusions.

## Conclusion

This study demonstrates that arousals show significant associations with SpO_2_ event architecture, with distinct patterns based on arousal timing relative to desaturation events. Pre-event arousals were associated with faster desaturation/resaturation rates and shorter durations, while within-event arousals were linked to lower nadirs, longer durations, and larger desaturation areas. These associations were amplified in the presence of respiratory events, particularly apnoeas, and showed significant sex-based differences. The distinct patterns observed in SpO_2_ parameters based on arousaltiming could contribute to compensatory biomarkers for arousal detection when EEG is not available, addressing a critical need in limited channel sleep studies. Future research may focus on developing and validating algorithms that incorporate these SpO_2_ patterns for automated arousal detection in limited channel settings, potentially improving diagnostic capabilities for sleep disorders in home-based testing environments.

## Supplementary Information

Below is the link to the electronic supplementary material.

Supplementary Material 1 (DOCX 23.1 KB)

## Data Availability

The data that support the findings of this study are available from the National Sleep Research Resource (NSRR). Restrictions apply to the availability of these data, which were used under license for this study. Data are available at https://sleepdata.org/datasets/shhs with the permission of the NSRR.

## Abbreviations

ABOSA: Automated blood oxygen saturation analysis
BMI: Body mass index
CI: Confidence interval
CO_2_: Carbon dioxide
EEG: Electroencephalography
IQR: Interquartile range
OSA: Obstructive sleep apnoea
SHHS: Sleep heart health study
SpO_2_: Oxygen saturation

## References

[1] Pahari P, Korkalainen H, Karhu T, Rissanen M, Arnardottir ES, Hrubos-Strøm H et al Obstructive sleep apnea-related intermittent hypoxaemia is associated with impaired vigilance. J Sleep Res e13803. 10.1111/jsr.13803 36482788

[2] Kainulainen S, Duce B, Korkalainen H, Oksenberg A, Leino A, Arnardottir ES et al (2020) Severe desaturations increase psychomotor vigilance task-based median reaction time and number of lapses in obstructive sleep apnoea patients. Eur Respir J 55(4):1901849. 10.1183/13993003.01849-2019 PMC7142879 32029446

[3] Kainulainen S, Töyräs J, Oksenberg A, Korkalainen H, Sefa S, Kulkas A et al (2019) Severity of desaturations reflects OSA-related daytime sleepiness better than AHI. J Clin Sleep Med 15(8):1135–42. 10.5664/jcsm.7806 PMC6707054 31482835

[4] Nikkonen S, Korkalainen H, Kainulainen S, Myllymaa S, Leino A, Kalevo L et al (2020) Estimating daytime sleepiness with previous night electroencephalography, electrooculography, and electromyography spectrograms in patients with suspected sleep apnea using a convolutional neural network. Sleep 43(12):zsaa106. 10.1093/sleep/zsaa106 PMC7734478 32459856

[5] Fanfulla F, Pinna GD, Marrone O, D’Artavilla Lupo N, Arcovio S, Bonsignore MR et al (2021) Determinants of sleepiness at wheel and missing accidents in patients with obstructive sleep apnea. Front Neurosci 15:656203 10.3389/fnins.2021.656203 PMC8076750 33927591

[6] Sabil A, Bignard R, Gervès-Pinquié C, Philip P, Le Vaillant M, Trzepizur W et al (2021) Risk factors for sleepiness at the wheel and sleep-related car accidents among patients with obstructive sleep apnea: data from the French Pays de la Loire sleep cohort. Nat Sci Sleep 13:1737–46. 10.2147/NSS.S328774 PMC8502051 34675722

[7] Oldenburg O, Wellmann B, Buchholz A, Bitter T, Fox H, Thiem U et al (2016) Nocturnal hypoxaemia is associated with increased mortality in stable heart failure patients. Eur Heart J 37(21):1695–1703. 10.1093/eurheartj/ehv624 26612581

[8] Azarbarzin A, Sands SA, Stone KL, Taranto-Montemurro L, Messineo L, Terrill PI et al (2019) The hypoxic burden of sleep apnoea predicts cardiovascular disease-related mortality: the Osteoporotic Fractures in Men Study and the Sleep Heart Health Study. Eur Heart J 40(14):1149–57. 10.1093/eurheartj/ehy624 PMC6451769 30376054

[9] Terrill PI (2020) A review of approaches for analysing obstructive sleep apnoea-related patterns in pulse oximetry data. Respirology 25(5):475–485. 10.1111/resp.13635 31246376

[10] Howarth TP, Karhu T, Kainulainen S, Chen X, Mahamid A, Töyräs J et al (2023) Oxygen resaturation rate is significantly associated with objectively assessed excessive daytime sleepiness in suspected obstructive sleep apnoea patients. Sleep Med 107:171–8. 10.1016/j.sleep.2023.04.027 37187080

[11] Howarth TP, Sillanmäki S, Karhu T, Rissanen M, Islind AS, Hrubos-Strøm H et al (2024) Nocturnal oxygen resaturation parameters are associated with cardiorespiratory comorbidities. Sleep Med 118:101–112. 10.1016/j.sleep.2024.03.047 38657349

[12] Howarth TP, Hietakoste S, Ebrahimian S, Rissanen M, Kainulainen S, Karhu T (2025) The interaction between comorbidities and sleep stages influences oxygen re-saturation characteristics. J Sleep Res e14459. 10.1111/jsr.14459 39905694

[13] Howarth TP, Karhu T, Leppänen T, Saad HB, Bahammam A, Nikkonen S (2026) Five-year longitudinal changes in oxygen saturation parameters in obstructive sleep apnoea: impact of age, body mass index, and comorbidities. Sleep Med 143:108866. 10.1016/j.sleep.2026.108866 41843971

[14] Garbarino S, Bragazzi NL (2024) Revolutionizing sleep health: the emergence and impact of personalized sleep medicine. J Pers Med. 10.3390/jpm14060598 PMC11204813 38929819

[15] Malhotra A, Ayappa I, Ayas N, Collop N, Kirsch D, Mcardle N et al (2021) Metrics of sleep apnea severity: beyond the apnea-hypopnea index. Sleep 44(7):zsab030. 10.1093/sleep/zsab030 PMC8271129 33693939

[16] Eckert DJ, Younes MK (2014) Arousal from sleep: implications for obstructive sleep apnea pathogenesis and treatment. J Appl Physiol 116(3):302–313. 10.1152/japplphysiol.00649.2013 23990246

[17] Kim JB, Seo BS, Kim JH (2019) Effect of arousal on sympathetic overactivity in patients with obstructive sleep apnea. Sleep Med 62:86–91. 10.1016/j.sleep.2019.01.044 30975558

[18] Amatoury J, Jordan AS, Toson B, Nguyen C, Wellman A, Eckert DJ (2018) New insights into the timing and potential mechanisms of respiratory-induced cortical arousals in obstructive sleep apnea. Sleep. 10.1093/sleep/zsy160 PMC6231527 30137568

[19] Souza G, Stornetta R, Stornetta D, Abbott S, Guyenet P (2019) Contribution of the retrotrapezoid nucleus and carotid bodies to hypercapnia- and hypoxia-induced arousal from sleep. J Neurosci. 10.1523/JNEUROSCI.1268-19.2019 PMC6891059 31641048

[20] Luukinen M, Pitkänen H, Leppänen T, Töyräs J, Islind AS, Kainulainen S et al (2024) Variation in the photoplethysmogram response to arousal from sleep depending on the cause of arousal and the presence of desaturation. IEEE J Transl Eng Health Med 12:328. 10.1109/JTEHM.2024.3349916 PMC10914203 38444399

[21] Yan J, Zhang X, Yen CW, Sun L, Yu E, Luo Y (2016) Role of electroencephalogram and oxygen saturation in the induction mechanism of arousal for obstructive sleep apnea-hypopnea syndrome patients. Biol Rhythm Res 47(4):483–495. 10.1080/09291016.2016.1141774

[22] Bonnet MH, Arand DL (2007) EEG Arousal Norms by Age. J Clin Sleep Med 03(03):271–274. 10.5664/jcsm.26796 PMC2564772 17561594

[23] Rosen IM, Kirsch DB, Chervin RD, Carden KA, Ramar K, Aurora RN et al (2017) Clinical use of a home sleep apnea test: an American Academy of Sleep Medicine position statement. J Clin Sleep Med 13(10):1205–7. 10.5664/jcsm.6774 PMC5612637 28942762

[24] Zan H, Yildiz A (2023) Multi-task learning for arousal and sleep stage detection using fully convolutional networks. J Neural Eng 20(5):056034. 10.1088/1741-2552/acfe3a 37769664

[25] Rad AB, Zabihi M, Zhao Z, Gabbouj M, Katsaggelos AK, Särkkä S (2019) Automated Polysomnography Analysis for Detection of Non-Apneic and Non-Hypopneic Arousals using Feature Engineering and a Bidirectional LSTM Network [Internet]. arXiv; [cited 2026 May 13]. Available from: 10.48550/arXiv.1909.02971 http://arxiv.org/abs/1909.02971

[26] Zhang GQ, Cui L, Mueller R, Tao S, Kim M, Rueschman M et al (2018) The National Sleep Research Resource: towards a sleep data commons. J Am Med Inform Assoc 25(10):1351–8. 10.1093/jamia/ocy064 PMC6188513 29860441

[27] Quan SF, Howard BV, Iber C, Kiley JP, Nieto FJ, O’Connor GT et al (1997) The Sleep Heart Health Study: design, rationale, and methods. Sleep 20(12):1077–1085 9493915

[28] Mueller R Sleep Heart Health Study [Internet]. [cited 2026 May 16]. Sleep Data - National Sleep Research Resource - NSRR. Available from: https://sleepdata.org/

[29] Karhu T, Leppänen T, Töyräs J, Oksenberg A, Myllymaa S, Nikkonen S (2022) ABOSA - freely available automatic blood oxygen saturation signal analysis software: structure and validation. Comput Methods Programs Biomed 226:107120. 10.1016/j.cmpb.2022.107120 36152624

[30] Karhu T, Leppänen T, Töyräs J, Oskenberg A, Myllymaa S, Nikkonen S (2022) Automatic oxygen saturation signal analysis software [Internet]. Available from: https://zenodo.org/record/6962129#.Y4W3dHZBxD8 10.1016/j.cmpb.2022.107120 36152624

[31] Rolon RE, Gareis IE, Larrateguy LD, Di Persia LE, Spies RD, Rufiner HL (2020) Automatic scoring of apnea and hypopnea events using blood oxygen saturation signals. Biomed Signal Process Control 62:102062. 10.1016/j.bspc.2020.102062

[32] Mueller R Sleep Heart Health Study – 6 626 Mop Rules For Assigning Sleep Stages - Sleep Data - National Sleep Research Resource - NSRR [Internet]. [cited 2026 May 14]. Available from: https://sleepdata.org/datasets/shhs/pages/mop/6-626-mop-rules-for-assigning-sleep-stages.md

[33] Mueller R Sleep Heart Health Study – 6 623 Mop Eeg Arousal - Sleep Data - National Sleep Research Resource - NSRR [Internet]. [cited 2026 May 14]. Available from: https://sleepdata.org/datasets/shhs/pages/mop/6-623-mop-eeg-arousal.md

[34] Leppänen T, Kulkas A, Oksenberg A, Duce B, Mervaala E, Töyräs J (2018) Differences in arousal probability and duration after apnea and hypopnea events in adult obstructive sleep apnea patients. Physiol Meas 39(11):114004. 10.1088/1361-6579/aae42c 30251964

[35] Zitting KM, Lockyer BJ, Azarbarzin A, Sands SA, Wang W, Wellman A et al (2023) Association of cortical arousals with sleep-disordered breathing events. J Clin Sleep Med 19(5):899–912. 10.5664/jcsm.10492 PMC10152355 36708264

[36] Mueller R Sleep Heart Health Study – 6 627 Mop Scoring Respiratory Events - Sleep Data - National Sleep Research Resource - NSRR [Internet]. [cited 2026 May 14]. Available from: https://sleepdata.org/datasets/shhs/pages/mop/6-627-mop-scoring-respiratory-events.md

[37] Bowerman C, Bhakta NR, Brazzale D, Cooper BR, Cooper J, Gochicoa-Rangel L et al (2023) A race-neutral approach to the interpretation of lung function measurements. Am J Respir Crit Care Med 207(6):768–74. 10.1164/rccm.202205-0963OC 36383197

[38] Bonsignore MR, Saaresranta T, Riha RL (2019) Sex differences in obstructive sleep apnoea. Eur Respir Rev. 10.1183/16000617.0030-2019 PMC9488655 31694839

[39] Mann LM, Chan JS, Angus SA, Doherty CJ, Thompson BP, Foster GE et al (1985) Peripheral hypercapnic chemosensitivity in trained and untrained females and males during exercise. J Appl Physiol 133(6):1309–17. 10.1152/japplphysiol.00460.2022 36302156

[40] Drapeau A, Imhoff S, Labrecque L, Paquette M, Le Blanc O, Malenfant S et al (2020) Cerebrovascular carbon dioxide reactivity is influenced by sex despite comparable hemodynamic and respiratory chemosensitivity in young fit individuals. FASEB J 34(S1):1–1. 10.1096/fasebj.2020.34.s1.09463

[41] Sands SA, Edwards BA, Terrill PI, Taranto-Montemurro L, Azarbarzin A, Marques M et al (2018) Phenotyping Pharyngeal Pathophysiology using Polysomnography in Patients with Obstructive Sleep Apnea. Am J Respir Crit Care Med 197(9):1187–1197. 10.1164/rccm.201707-1435OC PMC6019932 29327943

[42] Dawson A, Avraam J, Nicholas CL, Kay A, Thornton T, Feast N et al (2023) Mechanisms underlying the prolonged activation of the genioglossus following arousal from sleep. Sleep 47(1):zsad202. 10.1093/sleep/zsad202 PMC10782491 37503934

[43] Deacon N, Malhotra A (2016) Potential protective mechanism of arousal in obstructive sleep apnea. J Thorac Dis 8(Suppl 7):S545-6. 10.21037/jtd.2016.07.43 PMC4990681 27606089

[44] Oliven R, Cohen G, Dotan Y, Somri M, Schwartz AR, Oliven A (2018) Alteration in upper airway dilator muscle coactivation during sleep: comparison of patients with obstructive sleep apnea and healthy subjects. J Appl Physiol (1985) 124(2):421–9. 10.1152/japplphysiol.01067.2016 PMC11735003 29191983

[45] Sands SA, Terrill PI, Edwards BA, Taranto Montemurro L, Azarbarzin A, Marques M et al (2018) Quantifying the arousal threshold using polysomnography in obstructive sleep apnea. Sleep 41(1):zsx183. 10.1093/sleep/zsx183 PMC5804982 29228393

[46] Huang EI, Huang SY, Lin YC, Lin CM, Lin CK, Hsu CY et al (2022) Respiratory arousals in patients with very severe obstructive sleep apnea and how they change after a non-framework surgery. Healthcare (Basel) 10(5):902. 10.3390/healthcare10050902 PMC9140339 35628039

[47] Shahrbabaki SS, Linz D, Hartmann S, Redline S, Baumert M (2021) Sleep arousal burden is associated with long-term all-cause and cardiovascular mortality in 8001 community-dwelling older men and women. Eur Heart J 42(21):2088. 10.1093/eurheartj/ehab151 PMC8197565 33876221

[48] Bonnet MH, Arand DL (2003) Clinical effects of sleep fragmentation versus sleep deprivation. Sleep Med Rev 7(4):297–310. 10.1053/smrv.2001.0245 14505597

[49] Ehrlich F, Sehr T, Brandt M, Schmidt M, Malberg H, Sedlmayr M et al (2024) State-of-the-art sleep arousal detection evaluated on a comprehensive clinical dataset. Sci Rep 14(1):16239. 10.1038/s41598-024-67022-9 PMC11247076 39004643

[50] Pitkänen H, Nikkonen S, Rissanen M, Islind AS, Gretarsdottir H, Arnardottir ES et al (2024) Multi-centre arousal scoring agreement in the Sleep Revolution. J Sleep Res 33(4):e14127. 10.1111/jsr.14127 38148632

[51] Azarbarzin A, Ostrowski M, Hanly P, Younes M (2014) Relationship between arousal intensity and heart rate response to arousal. Sleep 37(4):645. 10.5665/sleep.3560 PMC4044744 24899756

[52] Malatantis-Ewert S, Bahr K, Ding H, Ludwig K, Koirala n et al (2022) Huppertz t,. A Novel Quantitative Arousal-Associated EEG-Metric to Predict Severity of Respiratory Distress in Obstructive Sleep Apnea Patients. Front Physiol 13:885270. 10.3389/fphys.2022.885270 PMC9257225 35812317

[53] Zhang J, Mann DL, Leppänen T, Azarbarzin A, Sands SA, Töyräs J et al The Relative Contributions of Arousal Presence and Arousal Intensity to Post-Respiratory-Event Ventilation in Obstructive Sleep Apnea [Internet]. medRxiv; 2025 [cited 2026 Jun 10]. p. 2025.08.30.25334191. Available from: https://www.medrxiv.org/content/ 10.1111/jsr.70412 42522982

[54] Chouchou F, Pichot V, Barthélémy JC, Bastuji H, Roche F (2014) Cardiac sympathetic modulation in response to apneas/hypopneas through heart rate variability analysis. PLoS ONE 9(1):e86434. 10.1371/journal.pone.0086434 PMC3899280 24466093

[55] Jordan AS, McEvoy RD, Edwards JK, Schory K, Yang CK, Catcheside PG et al (2004) The influence of gender and upper airway resistance on the ventilatory response to arousal in obstructive sleep apnoea in humans. J Physiol 558(Pt 3):993–1004. 10.1113/jphysiol.2004.064238 PMC1665031 15218069

[56] Dutta R, Delaney G, Toson B, Jordan AS, White DP, Wellman A et al (2021) A novel model to estimate key obstructive sleep apnea endotypes from standard polysomnography and clinical data and their contribution to obstructive sleep apnea severity. Ann Am Thorac Soc 18(4):656–67. 10.1513/AnnalsATS.202001-064OC PMC8008997 33064953

[57] Huttunen R, Leppanen T, Duce B, Arnardottir ES, Nikkonen S, Myllymaa S et al (2023) A Comparison of Signal Combinations for Deep Learning-Based Simultaneous Sleep Staging and Respiratory Event Detection. IEEE Trans Biomed Eng 70(5):1704–1714. 10.1109/TBME.2022.3225268 36441886

[58] Casal R, Di Persia LE, Schlotthauer G (2021) Classifying sleep–wake stages through recurrent neural networks using pulse oximetry signals. Biomed Signal Process Control 63:102195. 10.1016/j.bspc.2020.102195

[59] Korkalainen H, Aakko J, Duce B, Kainulainen S, Leino A, Nikkonen S et al (2020) Deep learning enables sleep staging from photoplethysmogram for patients with suspected sleep apnea. Sleep 43(11):zsaa098. 10.1093/sleep/zsaa098 PMC7658638 32436942

[60] Tkacova R, McNicholas WT, Javorsky M, Fietze I, Sliwinski P, Parati G et al (2014) Nocturnal intermittent hypoxia predicts prevalent hypertension in the European Sleep Apnoea Database cohort study. Eur Respir J 44(4):931–941. 10.1183/09031936.00225113 25102963

[61] Punjabi NM, Newman AB, Young TB, Resnick HE, Sanders MH (2008) Sleep-disordered breathing and cardiovascular disease: an outcome-based definition of hypopneas. Am J Respir Crit Care Med 177(10):1150–1155. 10.1164/rccm.200712-1884 PMC2383996 18276938

[62] O’Driscoll DM, Meadows GE, Corfield DR, Simonds AK, Morrell MJ (2004) Cardiovascular response to arousal from sleep under controlled conditions of central and peripheral chemoreceptor stimulation in humans. J Appl Physiol Bethesda Md 1985 96(3):865–870. 10.1152/japplphysiol.00749 14578367

