# Supplementary material for: Differential associations of arousals and respiratory events on oxygen desaturation and resaturation parameters in sleep
Source: Sleep Breath. 2026 Sep 11;30(5):257. doi: 10.1007/s11325-026-03797-7 (PMC13569603; doi:10.1007/s11325-026-03797-7)
Supplement: Supplementary file 1 [file 11325_2026_3797_MOESM1_ESM.docx]

**Supplement 1.** Demographic and clinical overview of included participants

|  | **Total**  **(*n*=5,484)** | **Female**  **(*n*=2,799)** | **Male**  **(*n*=2,685)** |
| --- | --- | --- | --- |
| Age (years) | 63 (55, 72) | 63 (55, 73) | 63 (55, 71) |
| Sex (male) | 2685 (49%) | - | - |
| Caucasian | 4662 (85%) | 2346 (83.8%) | 2316 (86.3%) |
| BMI (kg/m^2^) (*n*=5,443) | 27.68 (24.84, 30.92) | 27.34 (24.23, 31.21) | 27.95 (25.39, 30.73) |
| Underweight BMI <18.5 | 32 (0.6%) | 26 (0.9%) | 6 (0.2%) |
| Normal weight 18.5 ≤ BMI < 25 | 1416 (26%) | 846 (30.5%) | 570 (21.4%) |
| Overweight 25 ≤ BMI < 30 | 2293 (42.1%) | 1013 (36.5%) | 1280 (48%) |
| Obese BMI ≥ 30 | 1702 (31.3%) | 889 (32%) | 813 (30.5%) |
| Ever smoker (*n*=5,448) | 2917 (53.5%) | 1216 (43.7%) | 1701 (63.8%) |
| FEV_1_ (% predicted) (*n*=4,852) | 97.32 (85.42, 108.09) | 98.72 (86.9, 109.63) | 95.95 (84.05, 106.7) |
| FVC (% predicted) (*n*=4,852) | 100 (89.02, 110.65) | 101.37 (90.17, 111.89) | 98.46 (87.96, 109.12) |
| FEV_1_/FVC (absolute) (*n*=4,852) | 0.76 (0.72, 0.8) | 0.77 (0.73, 0.81) | 0.75 (0.71, 0.8) |
| ESS (*n*=5,281) | 7 (4, 11) | 7 (4, 10) | 8 (5, 11) |
| Total sleep time (hours) | 7.12 (6.42, 7.71) | 7.21 (6.52, 7.81) | 7.03 (6.35, 7.61) |
| No comorbidity or medication | 784 (14.3%) | 333 (11.9%) | 451 (16.8%) |
| Arterial hypertension | 2714 (49.5%) | 1357 (48.5%) | 1357 (50.5%) |
| Lung disease | 769 (14%) | 443 (15.8%) | 326 (12.1%) |
| Myocardial infarction | 771 (14.1%) | 270 (9.6%) | 501 (18.7%) |
| Diabetes mellitus | 439 (8%) | 187 (6.7%) | 252 (9.4%) |
| Stroke | 247 (4.5%) | 124 (4.4%) | 123 (4.6%) |
| Heart failure | 216 (3.9%) | 95 (3.4%) | 121 (4.5%) |
| Hormone therapy | 843 (15.4%) | 839 (30%) | 4 (0.1%) |
| Mental health medication | 602 (11%) | 392 (14%) | 210 (7.8%) |
| Data displayed as median (IQR) or number (%)  **Abbreviations**: **BMI**, Body mass index; **ESS**, Epworth sleepiness scale; **FEV_1_**, Forced expiratory volume in one second; **FVC**, Forced vital capacity; **IQR**, Interquartile range. | | | |

**Supplement 2.** Effect of sex (against reference of female participants) in fully adjusted models for desaturation and resaturation parameters stratified by respiratory event.

|  | No respiratory event | Hypopnoea | Apnoea |
| --- | --- | --- | --- |
| Pre-event SpO_2_ value | -0.14 (-0.2, -0.08)* | -0.1 (-0.14, -0.07)* | -0.04 (-0.09, 0.01) |
| Saturation event nadir | 0.02 (0, 0.05) | -0.04 (-0.06, -0.02)* | -0.12 (-0.16, -0.07)* |
| Post-event SpO_2_ value | -0.07 (-0.11, -0.04)* | -0.05 (-0.07, -0.03)* | -0.03 (-0.07, 0) |
| Desaturation duration (seconds) | 0.2 (0.13, 0.26)* | 0.14 (0.11, 0.17)* | 0.2 (0.15, 0.24)* |
| Desaturation depth (% point change) | -0.03 (-0.06, 0.01) | 0.05 (0.02, 0.07)* | 0.14 (0.09, 0.2)* |
| Desaturation rate (%/second) | -0.21 (-0.27, -0.14)* | -0.11 (-0.14, -0.08)* | -0.15 (-0.19, -0.1)* |
| Desaturation area (%s) | 0.09 (0.06, 0.13)* | 0.12 (0.1, 0.15)* | 0.22 (0.17, 0.28)* |
| Resaturation duration (seconds) | 0.15 (0.09, 0.22)* | 0.08 (0.04, 0.11)* | 0.02 (-0.02, 0.05) |
| Resaturation depth (% point change) | -0.05 (-0.08, -0.03)* | -0.04 (-0.05, -0.02)* | -0.02 (-0.05, 0) |
| Resaturation rate (%/second) | -0.13 (-0.17, -0.1)* | -0.08 (-0.1, -0.06)* | -0.05 (-0.09, -0.02) |
| Resaturation area (%s) | 0.1 (0.05, 0.15)* | 0.06 (0.03, 0.08)* | 0.03 (0, 0.05) |
| Parameters displayed as beta (95% confidence interval) obtained via multivariate mixed effects model regression.  Models were adjusted for age, body mass index, forced expiratory volume in one second, sleep stage, smoking history, presence of arousal, presence of respiratory event and presence/history of arterial hypertension, lung disease, heart failure, diabetes mellitus, myocardial infarction, stroke, use of hormone therapy and mental health medications. Desaturation duration, depth, rate, and area were additionally adjusted for pre-event oxygen saturation (SpO_2_). Resaturation duration, depth, rate, and area were additionally adjusted for pre-event SpO_2_ value and event nadir.  * indicates Bonferroni corrected p-value <0.05 | | | |

**Supplement 3.** Effect of arousals (against reference of No Arousal) in fully adjusted models for standardised desaturation and resaturation parameters stratified by respiratory event and arousal timing

| **Pre-event arousal** | No respiratory event | Hypopnoea | Apnoea |
| --- | --- | --- | --- |
| Pre-event SpO_2_ value | -0.04 (-0.15, 0.06) | -0.1 (-0.14, -0.06)* | -0.58 (-0.62, -0.53)* |
| Saturation event nadir | 0 (-0.06, 0.06) | 0.04 (0.01, 0.07) | 0.16 (0.11, 0.2)* |
| Post-event SpO_2_ value | -0.02 (-0.11, 0.07) | 0.26 (0.22, 0.29)* | 0.39 (0.35, 0.43)* |
| Desaturation duration | -0.25 (-0.39, -0.11)* | -0.34 (-0.39, -0.29)* | -0.36 (-0.4, -0.31)* |
| Resaturation duration | -0.22 (-0.38, -0.07) | -0.23 (-0.29, -0.17)* | -0.12 (-0.16, -0.08)* |
| Desaturation depth | 0 (-0.08, 0.07) | -0.05 (-0.09, -0.02) | -0.19 (-0.25, -0.13)* |
| Resaturation depth | -0.01 (-0.07, 0.05) | 0.18 (0.16, 0.2)* | 0.27 (0.24, 0.3)* |
| Desaturation rate | 0.42 (0.27, 0.56)* | 0.54 (0.5, 0.58)* | 0.58 (0.54, 0.63)* |
| Resaturation rate | 0.09 (-0.01, 0.18) | 0.35 (0.31, 0.38)* | 0.35 (0.31, 0.4)* |
| Desaturation area | -0.08 (-0.16, 0) | -0.19 (-0.23, -0.15)* | -0.27 (-0.33, -0.2)* |
| Resaturation area | -0.11 (-0.24, 0.01) | -0.08 (-0.13, -0.03) | 0 (-0.05, 0.04) |
| **Within event arousal** |  |  |  |
| Pre-event SpO_2_ value | 0.11 (0.07, 0.14)* | 0.1 (0.09, 0.11)* | 0.15 (0.14, 0.16)* |
| Saturation event nadir | -0.09 (-0.11, -0.07)* | -0.2 (-0.2, -0.19)* | -0.4 (-0.41, -0.39)* |
| Post-event SpO_2_ value | 0.21 (0.18, 0.24)* | 0.35 (0.34, 0.36)* | 0.23 (0.22, 0.24)* |
| Desaturation duration | 0.65 (0.61, 0.7)* | 0.33 (0.32, 0.34)* | 0.38 (0.37, 0.39)* |
| Resaturation duration | 0.35 (0.31, 0.4)* | 0.18 (0.16, 0.19)* | 0.08 (0.06, 0.09)* |
| Desaturation depth | 0.11 (0.09, 0.13)* | 0.24 (0.23, 0.25)* | 0.48 (0.47, 0.5)* |
| Resaturation depth | 0.15 (0.13, 0.16)* | 0.25 (0.24, 0.25)* | 0.16 (0.15, 0.17)* |
| Desaturation rate | -0.62 (-0.66, -0.57)* | -0.15 (-0.16, -0.14)* | -0.07 (-0.08, -0.06)* |
| Resaturation rate | -0.06 (-0.09, -0.03)* | 0.1 (0.09, 0.1)* | 0.1 (0.09, 0.11)* |
| Desaturation area | 0.24 (0.22, 0.27)* | 0.27 (0.26, 0.28)* | 0.51 (0.49, 0.52)* |
| Resaturation area | 0.3 (0.26, 0.33)* | 0.29 (0.28, 0.31)* | 0.13 (0.12, 0.14)* |
| Parameters displayed as beta (95% confidence interval) obtained via multivariate mixed effects model regression  Models were adjusted for age, BMI, FEV_1_, sleep stage, smoking history, presence of arousal, presence of respiratory event and presence/history of HTN, lung disease, heart failure, diabetes, myocardial infarction, stroke, use of hormone therapy and mental health medications. Desaturation duration, depth, rate, and area were additionally adjusted for pre-event SpO_2_ value. Resaturation duration, depth, rate, and area were additionally adjusted for pre-event SpO_2_ value and event nadir.  * Indicates Bonferroni adjusted p-value <0.05 | | | |
